# Association between ADIPOQ G276T and C11377G polymorphisms and the risk of non‐alcoholic fatty liver disease: An updated meta‐analysis

**DOI:** 10.1002/mgg3.624

**Published:** 2019-03-05

**Authors:** Mengwei Liu, Shan Liu, Mengke Shang, Xiuping Liu, Yue Wang, Qian Li, Michael Mambiya, Luping Yang, Qian Zhang, Kaili Zhang, Fangfang Nie, Fanxin Zeng, Wanyang Liu

**Affiliations:** ^1^ Department of Nutrition and Food Hygiene School of Public Health China Medical University Shenyang P.R. China

**Keywords:** adiponectin gene, *ADIPOQ*, meta‐analysis, metabolism syndrome, non‐alcoholic fatty liver disease, polymorphism

## Abstract

**Background:**

Nonalcoholic fatty liver disease (NAFLD) is a significant contributor to global hepatic disorders. *ADIPOQ* gene single‐nucleotide polymorphisms have been associated with NAFLD susceptibility, but with inconsistent results across the studies. This study aimed to investigate the association between *ADIPOQ* polymorphisms (+276G>T, rs1501299 and −11377C>G, rs266729) and the risk of NAFLD.

**Methods:**

PubMed, Embase, Wanfang, Web of Science, and China National Knowledge Infrastructure databases were used to identify the relevant published literature. Statistical analyses were calculated with STATA 11.0 software and RevMan 5.2. Summary odds ratios (OR) and 95% confidence intervals (CIs) were generated to assess the strength of the associations.

**Results:**

Eleven relevant articles with a total of 3,644 participants (1,847 cases/1,797 controls) were included. Our meta‐analysis results revealed that *ADIPOQ* gene +276G>T polymorphism was not associated with NAFLD under various genetic models (allele model: OR = 0.99, 95% CI [0.69, 1.41]; dominant model: OR = 1.06, 95% CI [0.71, 1.58]; recessive model: OR = 0.83, 95% CI [0.42, 1.65]; homozygous model: OR = 0.86, 95% CI [0.38, 1.95]; heterozygous model: OR = 1.10, 95% CI [0.80, 1.53]; respectively). Moreover, no statistical significant association was found between +276G>T and NAFLD risk in the subgroups. *ADIPOQ* gene −11377C>G polymorphism significantly increased the risk of NAFLD (allele model: OR = 1.49, 95% CI [1.28, 1.75]; dominant model: OR = 1.64, 95% CI [1.35, 1.99]; recessive model: OR = 1.77, 95% CI [1.16, 2.70]; homozygous model: OR = 2.13, 95% CI [1.38, 3.28]; heterozygous model: OR = 1.58, 95% CI [1.29, 1.93]; respectively).

**Conclusion:**

*ADIPOQ* gene −11377C>G may be a risk factor for NAFLD, while there was no association between *ADIPOQ* gene +276G>T polymorphism and the risk of NAFLD. Further studies are needed to detect the relationship between these *ADIPOQ* polymorphisms and NAFLD.

## 1 INTRODUCTION

Nonalcoholic fatty liver (NAFLD) is one of the most common chronic liver diseases worldwide (Chalasani et al., 2018; Younossi et al., 2016). NAFLD is a prevalent metabolic liver disease, which is on the rise in the world (Bugianesi et al., 2002; Dyson et al., 2014). Up to one‐fifth of NAFLD patients will develop nonalcoholic steatohepatitis, which may further develop into liver fibrosis, cirrhosis and complications, including hepatocellular carcinoma (Charlton et al., 2011; Kacso, Trifa, Popp, & Kacso, 2012). Little is known about the underlying mechanism for the development and progress of NAFLD, however, it is a complex metabolic state in which both lifestyle and genetic factors are pathogenic factors.

*ADIPOQ* (OMIM: 605441) is a kind of adipose tissue specific cytokine secreted mainly by white adipose tissue, which plays an important role in regulating insulin sensitivity, glucose homeostasis and lipid metabolism (Bessone, Razori, & Roma, 2019; Fu, 2014). NAFLD is the main liver manifestation of metabolic syndrome (Marchesini et al., 1999; Socha et al., 2007; Yki‐Jarvinen, 2014). Not only is insulin resistance an independent risk factor for NAFLD severity, but obesity, Type 2 diabetes, dyslipidemia and hypertension are also major causes of NAFLD (Cai et al., 2005; Lindenmeyer & McCullough, 2018; Wong et al., 2008)**.** Therefore, we hypothesized that the gene coding for *ADIPOQ*, adiponectin, also known as AMP1 gene, located on chromosome 3q27, which is the susceptible locus for NAFLD. To date, in diverse populations, massive studies have explored the relationship between *ADIPOQ* gene single‐nucleotide polymorphisms (SNPs) and NAFLD risk, as rs1501299 and 266729 (Stefan, Haring, & Cusi, 2018). However, the results of these studies have been inconsistent, may be due to sample sizes, diverse ethnicity and so on. Therefore, we conducted a meta‐analysis to detect the associations between two *ADIPOQ* gene polymorphisms and NAFLD risk by previous studies.

## 2 MATERIALS AND METHODS

### 2.1 Literature search strategy

We used PubMed, Embase, Wanfang, Web of Science, and China National Knowledge Infrastructure databases to search for potentially relevant studies published before November, 2018, which focused on the associations of the two polymorphisms in the *ADIPOQ* gene (rs1501299, rs266729) with NAFLD susceptibility without language restrictions. The search strategy was based on a combination of the following terms: adiponectin or *ADIPOQ* or adipose most abundant gene transcript 1 or APM1 or rs1501299 or rs266729 AND nonalcoholic fatty liver disease or NAFLD or nonalcoholic steatohepatitis AND single‐nucleotide polymorphism or genetic polymorphism. We performed a manual search of references included in pertinent articles and reviews. If there was duplication of published literature by the same research group, the study with the larger sample size was selected.

### 2.2 Inclusion and exclusion criteria

Potentially relevant studies in our meta‐analysis have to meet following inclusion criteria: (a) case‐control studies focused on the association between the *ADIPOQ* gene+276G>T (NM_001177800.1:c.214+62G>T) and −11377C>G (NG_021140.1:g.4012C>G) and NAFLD risk, (b) every patient selected have to base on diagnostic criteria for NAFLD: persistently (at least 6 months) abnormal levels of aspartate aminotransferase and alanine aminotransferase, or evidence of fatty liver using ultrasound or other imaging techniques, with a daily alcohol consumption <20 g/day in men and <10 g/day in women; patients with infectious (such as hepatitis B virus and hepatitis C virus and HIV), viral, drug‐induced, auto‐immune hepatitis, and other serious diseases (including severe heart, lung, brain, or kidney diseases) were excluded, (c) the genotype distribution in control group should accord with Hardy–Weinberg equilibrium (HWE), (d) published data of allele and genotype frequencies must be explicit or could be calculated from the article text. Excluded articles should have the following characteristics: (a) case‐control studies without control information, (b) existing duplicate publication of data, (c) not enough data information form articles.

### 2.3 Data extraction

Original data from published studies were independently extracted by two investigators into a standardized form. Contested issues in data extraction were resolved through discussions and re‐inspection with the third investigator. The following information was collected: the first author's surname, year of publication, country, ethnicity, source of controls, number of cases and controls, genotyping methods, frequency of allele and genotype, HWE and Newcastle–Ottawa Scale (NOS) score.

### 2.4 Quality assessment

The methodological quality of each literature included in our analysis was estimated using the NOS, which scores of 1–3, 4–6 and 7–9 meaning low‐, intermediate‐, and high quality studies respectively in this meta‐analysis. Two authors independently evaluated the quality of selected articles. Differences were resolved by the third author or by discussion, until subsequent consensus was reached.

### 2.5 Statistical analysis

Summary odds ratios (ORs) with 95% confidence intervals (CIs) were calculated to quantitatively assess the association between *ADIPOQ* SNPs and NAFLD risk under five comparison models: the allele model, dominant model, recessive model, homozygous model, and heterozygous model. HWE in the control group was tested by chi‐square test (χ^2^‐test) for studies included in the current meta‐analysis. *Z*‐test was applied to examine the statistical significance of the pooled OR. Among‐studies heterogeneity was assessed using Cochran's *Q*‐test and *I* ^2^‐test. When *I* ^2^ > 50% or *p* < 0.05, the heterogeneity was deemed as significant, we selected the random‐effect model to calculate pooled ORs, on the contrary, the fixed‐effect mode was performed. Sensitivity analysis was performed by removing each individual studies sequentially or by deleting the outliers to assess the stability of the results. Begg's funnel plots were conducted to assess the significance of publication bias, and Egger's test was further supplemented (*p* < 0.05 was considered representation of statistically significant publication bias). Meta‐analysis was conducted using the Cochrane Collaboration RevMan 5.2 and Stata 11.0 software (Stata Corporation, College Station, TX).

## 3 RESULTS

### 3.1 Characteristics of the studies included

**Figure 1:**
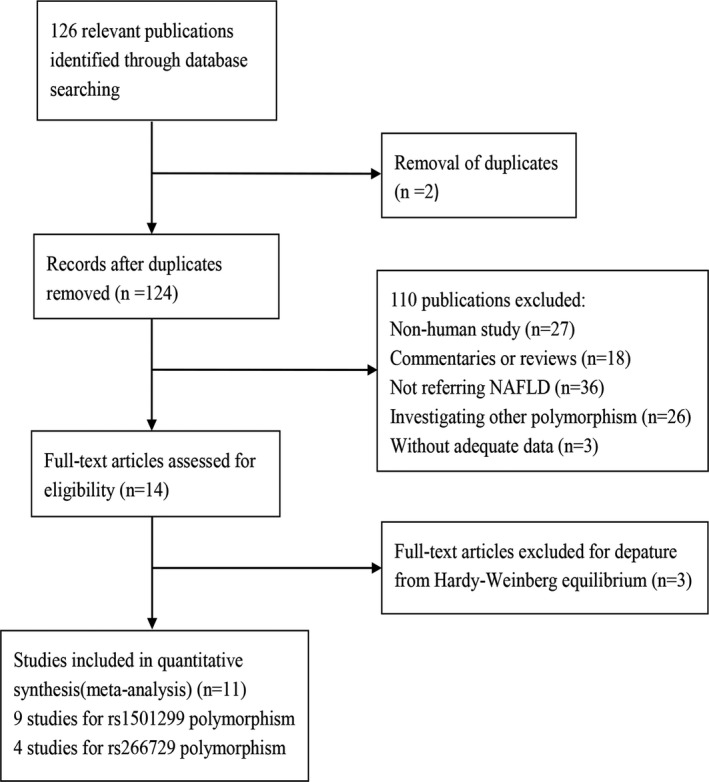
Flow diagram of the study search and selection process. NAFLD: nonalcoholic fatty liver disease

The search strategy retrieved 126 potentially relevant studies, by browsing the title and abstract, a total of 107 articles were excluded. Then two other articles were removed because of repeated publications. Three articles were excluded because of a lack of detailed genotype distribution information. Three articles were excluded due to distributions of genotypes in controls were not consistent with the HWE. Ultimately, we included eleven articles (Cheng, Jiang, Xin, An, & Xuan, 2015; Hashemi et al., 2013; He, Xu, Han, Chen, & Chen, 2017; Li, Li, Zhang, Zhong, & Shi, 2015; Mohseni, Moghbelinejad, & Najafipour, 2017; Musso et al., 2008; Tokushige et al., 2009; Wang et al., 2008; Wei, Li‐Qun, Xiao‐Ling, Jian, & Guo‐Yue, 2016; Ye, Yu, Wang, & Lu, 2014; Zhou et al., 2010), involving 1,847 cases and 1,797 controls in present meta‐analysis. The study selection outlined is shown in Figure 1. The characteristics of main studies are summarized in Table 1. There were 10 studies for *ADIPOQ* +276G>T, the features of these ten articles: nine studies focused on Asian ethnicities and one study was Caucasian populations, besides one study (Gong et al., 2013) that does not conform to HWE. So there are nine articles that meet the inclusion criteria finally. For −11377C>G polymorphism, six relevant studies were included in Table 1, among them, due to two studies (Gupta et al., 2012; Zhang, Guo, Qin, & Li, 2016) of the distribution of genotypes in controls were not consistent with the HWE. So, eligible studies were four on this related topic.

**Table 1:** Main characteristics of all eligible studies

First author (year)	Country	Ethnicity	Source of controls	Sample size (case/control)	Genotyping method	Case (genotype)			Control (genotype)			HWE^a^	NOS score
						GG	GT	TT	GG	GT	TT		
rs1501299													
Musso et al. (2008)	Italy	Caucasian	PB	70/70	PCR‐RFLP	17	51	2	38	29	3	0.381	9
Hashemi et al. (2013)	Iran	Asian	PB	83/93	Tetra ARMS‐PCR	42	39	2	53	38	2	0.104	9
He et al. (2017)	Chinese	Asian	HB	102/100	PCR‐RFLP	49	36	17	20	39	41	0.066	7
Li et al. (2015)	Chinese	Asian	HB	357/357	PCR‐RFLP	113	164	80	161	165	31	0.215	7
Mohseni et al. (2017)	Iran	Asian	PB	75/76	PCR‐sanger sequencing	33	32	10	39	28	9	0.268	8
Tokushige et al. (2009)	Japan	Asian	PB	119/115	AS‐PCR	67	47	4	59	47	9	0.932	9
Zhou et al. (2010)	Chinese	Asian	PB	106/106	PCR‐RFLP	68	29	9	50	39	17	0.057	7
Wang et al. (2008)	Chinese	Asian	HB	165/160	PCR‐RFLP	74	82	9	74	73	13	0.392	9
Zhang et al. (2016)	Chinese	Asian	HB	302/310	PCR‐RFLP	161	120	21	184	112	14	0.557	7
Gong et al. (2013)	Chinese	Asian	PB	255/405	PCR‐RFLP	155	75	25	201	153	51	**0.012**	6

						CC	CG	GG	CC	CG	GG		
rs266729													
Gupta et al. (2012)	India	Asian	HB	137/250	PCR	77	53	7	156	92	2	**0.003**	7
Cheng et al. (2015)	Chinese	Asian	HB	338/280	PCR‐sanger sequencing	158	149	31	164	102	14	0.715	8
Hashemi et al. (2013)	Iran	Asian	PB	83/93	Tetra ARMS‐PCR	27	53	3	49	41	3	0.107	8
Ye et al. (2014)	Chinese	Asian	HB	130/130	PCR‐RFLP	77	47	6	83	40	7	0.458	7
Zhang et al. (2016)	Chinese	Asian	HB	200/200	PCR‐RFLP	57	69	74	143	28	29	**0**	7
Zhang et al. (2016)	Chinese	Asian	HB	302/310	PCR‐RFLP	152	126	24	197	102	11	0.619	7

Notes

HB: hospital‐based; PB: population‐based; AS: allele‐specific; PCR‐RFLP: polymerase chain reaction restriction fragment length polymorphism; NOS: Newcastle–Ottawa Scale; ARMS‐PCR: amplification refractory mutation system; HWE: Hardy–Weinberg Equilibrium.

The numbers in bold values indicate that the genotype distribution of control group in certain studies do not accord with Hardy‐Weinberg equilibrium.

a *p* value for HWE test in controls.

### 3.2 Associations between +276G>T (rs1501299) polymorphism and NAFLD

**Figure 2:**
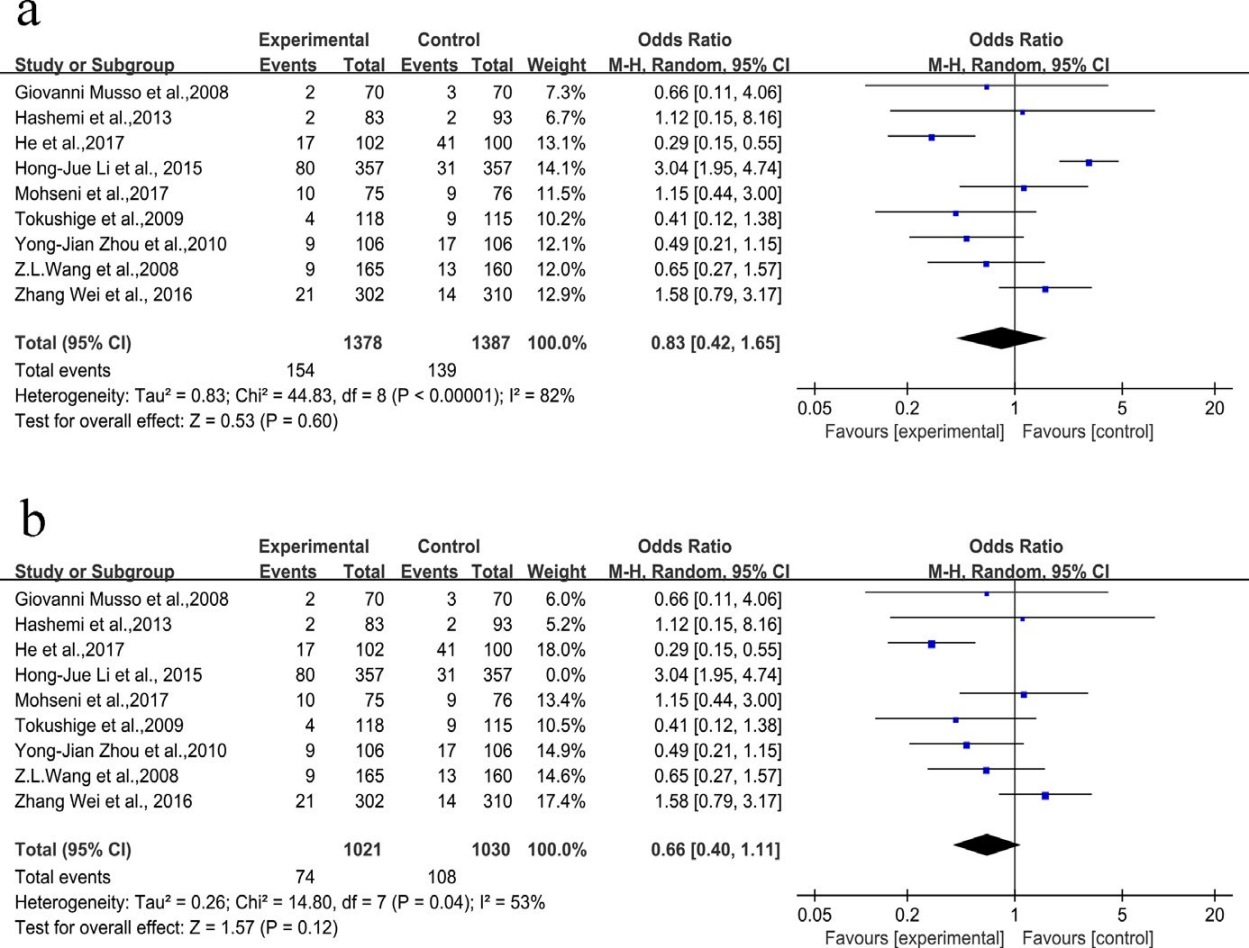
Forest plot for rs1501299 under recessive model (TT vs. GT+GG). (a) Pooled results, (b) results omitting Hong‐Jue Li et al. (2015)

We included nine articles published on the association between +276G>T polymorphism and NAFLD risk. We found that no significant associations between this polymorphism and NAFLD risk (alleles model: OR = 0.99, 95% CI [0.69, 1.41]; dominant model: OR = 1.06, 95% CI [0.71, 1.58]; recessive model: OR = 0.83, 95% CI [0.42, 1.65]; homozygous model: OR = 0.86, 95% CI [0.38, 1.95]; heterozygous: OR = 1.10, 95% CI [0.80, 1.53], respectively, Table 2) based on combined results from all studies. Yet significant heterogeneity was observed in overall comparisons. To estimate the source of heterogeneity, we performed sensitivity analyses though omitting each individual study in turn. For recessive model, the significant heterogeneity decreased though excluded the study by Li et al. (2015), it means that this study might be a part of source of heterogeneity (Figure 2). While sensitivity analysis failed to fully explain the source of heterogeneity. So to further explore the heterogeneity of five genetic models, we adopted subgroup analysis (Table 3). Our analyses showed that no associations between +276G>T polymorphism and NAFLD risk were observed in the subgroups in five genetic models.

**Table 2:** Meta‐analysis results of the associations between rs1501299 and rs266729 polymorphisms in *ADIPOQ* gene and non‐alcoholic fatty liver disease risk

Genetic model	No. of studies	OR [95% CI]	*p*_meta‐analysis_	*I* ^2^ (%)	*p* aheterogeneity	Statistical method
rs1501299						
T vs. G	9	0.99 [0.69, 1.41]	0.94	89	0	Random
GT+TT vs.GG	9	1.06 [0.71, 1.58]	0.78	84	0	Random
TT vs. GT+GG	9	0.83 [0.42, 1.65]	0.6	82	0	Random
TT vs. GG	9	0.86 [0.38, 1.95]	0.71	86	0	Random
GT vs. GG	9	1.10 [0.80, 1.53]	0.55	73	0.0002	Random
rs266729						
G vs. C	4	1.49 [1.28, 1.75]	0	0	0.51	Fixed
CG+GG vs. CC	4	1.64 [1.35, 1.99]	0	0	0.45	Fixed
GG vs. CG+CC	4	1.77 [1.16, 2.70]	0.009	0	0.46	Fixed
GG vs. CC	4	2.13 [1.38, 3.28]	0.0006	0	0.44	Fixed
CG vs. CC	4	1.58 [1.29, 1.93]	0.0001	0	0.51	Fixed

OR: odd ratio; CI: confidence interval.

a *p* value for between‐study heterogeneity based on *Q* test.

**Table 3:** Subgroup analysis for rs1501299 polymorphisms in *ADIPOQ* gene and non‐alcoholic fatty liver disease risk

Study group	Study numbers	T vs. G		GT+TT vs. GG		TT vs. GT+GG		TT vs. GG		GT vs. GG	
		OR [95% CI]	*I* ^2^ (%)/Ph	OR [95% CI]	*I* ^2^ (%)/Ph	OR [95% CI]	*I* ^2^ (%)/Ph	OR [95% CI]	*I* ^2^ (%)/Ph	OR [95% CI]	*I* ^2^ (%)/Ph
Total	9	0.99 [0.69, 1.41]	89/0.000	1.06 [0.71, 1.58]	84/0.000	0.83 [0.42, 1.65]	82/0.000	0.86 [0.38, 1.95]	86/0.000	1.10 [0.80, 1.53]	73/0.000
Enthicity											
Caucasian	1	1.91 [1.14, 3.18]	a	3.70 [1.80, 7.61]	a	0.66 [0.11, 4.06]	a	1.49 [0.23, 9.75]	a	3.93 [1.89, 8.17]	a
Asian	8	0.92 [0.63, 1.34]	89.4/0.000	0.93 [0.63, 1.38]	82.4/0.000	0.86 [0.41, 1.75]	84.3/0.000	0.82 [0.34, 1.96]	87.9/0.000	0.99 [0.75, 1.32]	62/0.01
Region											
Italy	1	1.91 [1.14, 3.18]	a	3.70 [1.80, 7.61]	a	0.66 [0.11, 4.06]	a	1.49 [0.23, 9.75]	a	3.93 [1.89, 8.17]	a
Iran	2	1.12 [0.86, 1.71]	0/0.955	1.32 [0, 85, 2.03]	0/0.935	1.14 [0.48, 2.71]	0/0.986	1.30 [0.53, 3.22]	0/0.972	1.32 [0.84, 2.08]	0/0.928
Chinese	5	0.85 [0.49, 1.47]	93.7/0.000	0.84 [0.47, 1.48]	89.4/0.000	0.87 [0.33, 2.30]	90.4/0.000	0.80 [0.24, 2.62]	92.7/0.000	0.90 [0.60, 1.36]	76.6/0.002
Japan	1	0.77 [0.51, 1.17]	a	0.80 [0.48, 1.34]	a	0.41 [0.12, 1.38]	a	0.39 [0.11, 1.34]	a	0.88 [0.52, 1.50]	a
Source of control											
PB	5	1.02 [0.67, 1.55]	75.7/0.002	1.17 [0.64, 2.16]	80.8/0.000	0.66 [0.39, 1.11]	0/0.626	0.69 [0.37, 1.29]	19.3/0.292	1.24 [0.68, 2.24]	77.8/0.001
HB	4	0.95 [0.52, 1.73]	94.2/0.000	0.95 [0.51, 1.74]	89.5/0.000	0.99 [0.32, 3.07]	91.9/0.000	0.96 [0.24, 3.74]	93.7/0.000	1.01 [0.67, 1.54]	74.6/0.008
Genotyping method											
PCR‐RFLP	6	0.96 [0.59‐1.58]	92.6/0.000	1.03 [0.59, 1.82]	89.5/0.000	0.84 [0.34, 2.06]	88.1/0.000	0.86 [0.29, 2.55]	90.8/0.000	1.08 [0.68, 1.72]	82.4/0.000
Non‐PCR‐RFLP	3	1.02 [0.75, 1.38]	25.5/0.261	1.07 [0.76, 1.50]	3.3/0.355	0.81 [0.40, 1.63]	0/0.407	0.84 [0.37, 1.91]	16.4/0.302	1.11 [0.79, 1.57]	0/0.526

*Notes*. CI: confidence interval; OR: odds ratio; Ph: *p* value for heterogeneity from *Q*‐test; *I* ^2^: the proportion of the total variation across studies due to heterogeneity; HB: hospital‐based; PB: population‐based; PCR‐RFLP: polymerase chain reaction restriction fragment length polymorphism.

a No heterogeneity was observed for only one study.

### 3.3 Associations between −11377C>G (rs266729) polymorphism and NAFLD

**Figure 3:**
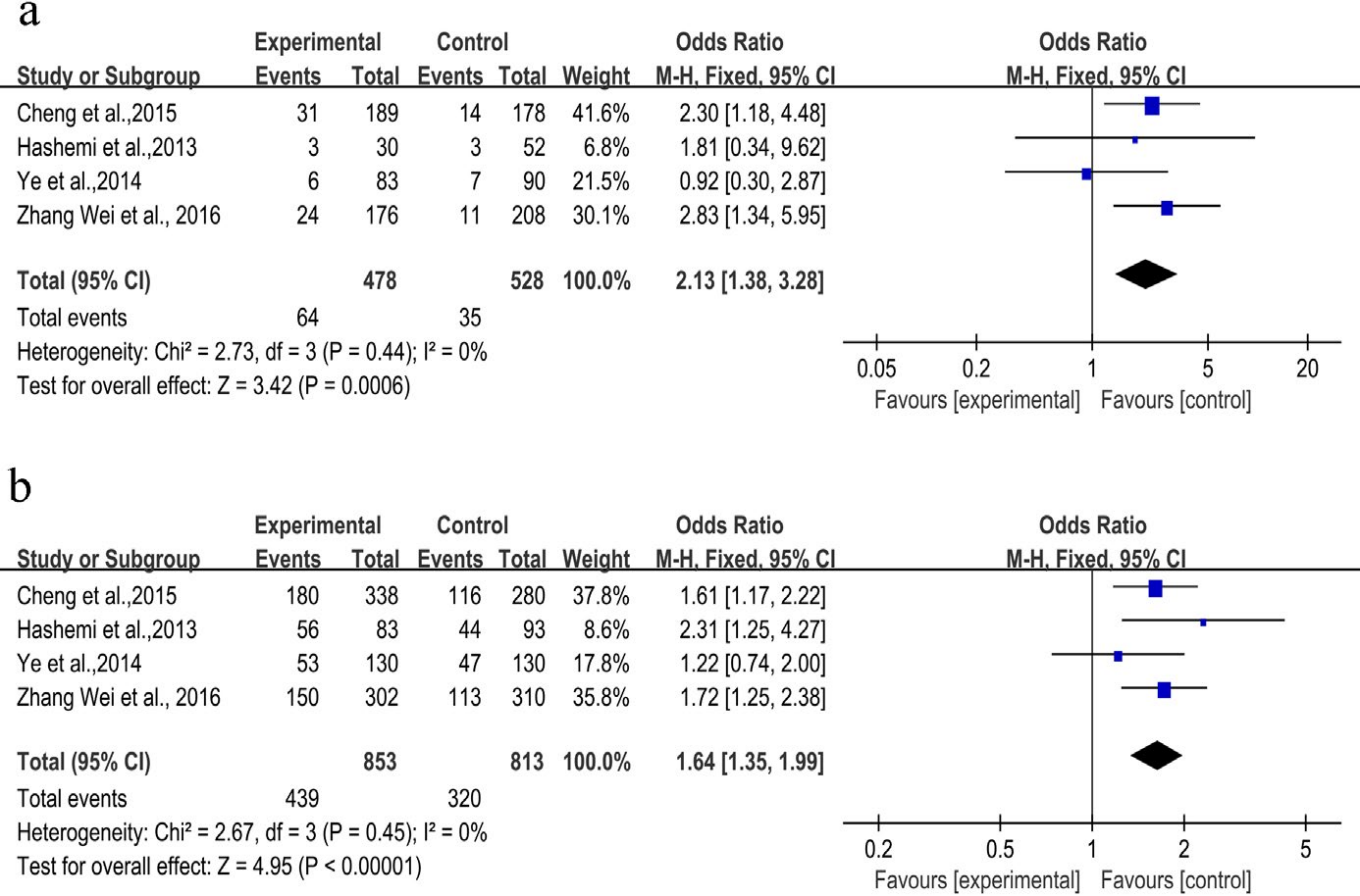
Forest plot represents the association between the rs266729 polymorphism and the risk of nonalcoholic fatty liver disease. (a) the homozygous model (GG vs. CC), (b) dominant model (CG+GG vs. CC)

Table 2 showed the pooled results of this meta‐analysis for −11377C>G polymorphism and NAFLD involving four articles published. All participator came from Asian population. A significant association under five genetic models analysis was found (alleles model: OR = 1.49, 95% CI [1.28, 1.75]; dominant model: OR = 1.64, 95% CI [1.35, 1.99]; recessive model: OR = 1.77, 95% CI [1.16, 2.70]; homozygous model: OR = 2.13, 95% CI [1.38, 3.28]; heterozygous: OR = 1.58, 95% CI [1.29, 1.93], Table 2). Meanwhile, heterogeneity test across the four studies showed no statistical significance (*I* ^2^ = 0%, Figure 3).

### 3.4 Publication bias

**Figure 4:**
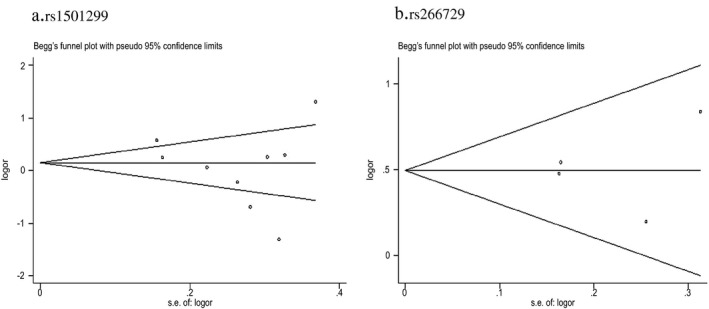
Begg's funnel plot of publication biases on the relationships between rs1501299 (a) and rs266729 (b) susceptibility with risk of non‐alcoholic fatty liver disease under dominant model. Each point represents a separate study for the indicated association. Log [OR], natural logarithm of the odds ratio, vertical line, means effect size

Begg's funnel plot was conducted to evaluate potential publication bias among included studies. No publication bias was apparent concerning relationship between rs266729 polymorphisms and NAFLD risk, as same as rs1501299 (Figure 4).

## 4 DISCUSSION

The worldwide prevalence of NAFLD is on the rise, and it is rapidly becoming the most common cause of chronic liver disease affecting 25% of the world's population (Chalasani et al., 2018; Charlton et al., 2011; Townsend & Newsome, 2016). The characteristics of metabolic syndrome (Mets) are not only very common in NAFLD patients, but also the components of Mets increase the risk of NAFLD, such as existing etiology: obesity, Type 2 diabetes, hypertension, and dyslipidemia and emerging elements: sleep apnea, colorectal cancer, osteoporosis, psoriasis, endocrinopathies, and polycystic ovary syndrome (Nasr, Ignatova, Kechagias, & Ekstedt, 2018; Stefan, Kantartzis, & Haring, 2008). The pathogenesis of NAFLD is particularly complex, since it involves interactions between genetic and environmental factors, many of which have been indistinct (Heid et al., 2006). *ADIPOQ* is expressed and secreted completely from adipocytes and has been identified as a cytokine with anti‐diabetes, anti‐inflammatory, and anti‐atherosclerosis properties (Fu, 2014). In addition, previous studies have demonstrated that *ADIPOQ* gene polymorphism may be influence plasma adiponectin concentration (Heid et al., 2006; Kadowaki et al., 2006; Maeda et al., 2001; Menzaghi et al., 2002). Significantly up‐regulated adiponectin expression in white adipose tissue leads to increased serum adiponectin concentrations (Targher et al., 2006). Low adiponectin level is closely related to the severity of liver histology, thus further supporting the hypothesis that adiponectin may be involved in the development of NAFLD (Vernon, Baranova, & Younossi, 2011). Although, several studies’ findings concerning relationship between −11377C>G and +276G>T polymorphism and NAFLD risk have been contradictory. Furthermore, previous meta‐analysis had its own limitations, we performed an updated meta‐analysis which comprehensively estimated the correlation between *ADIPOQ* polymorphisms and NAFLD risk. This present meta‐analysis included eligible eleven case‐control studies that were included nine studies for rs1501299 and four studies for rs266729, involving 1847 cases and 1797 controls. In general, for +276G>T polymorphism, we found that no significant association with NAFLD risk based on pooled results from all eligible studies. This conclusion might be contributed to inadequate adjustment for confounding factors, such as ethnicity, source of control, genotyping methods. By subgroup analysis, our analyses showed that no associations between this polymorphism and NAFLD risk were observed in the subgroup of country (Iran) in five genetic models, and the same result in the nonpolymerase chain reaction restriction fragment length polymorphism subgroup, no association was also detected in five genetic models. When excluded the study by Li et al. (2015), the heterogeneity drop in recessive model. Although sensitivity analysis does not explain the source of heterogeneity very well, it suggests that this particular study might be a part of a heterogeneous source. Besides, no publication bias was identified between studies, and thus we do not feel that it impacts the results.

And a significantly increased risk was discovered for relationship between −11377C>G polymorphism and NAFLD risk in different genetic models. Due to four articles focused on Asians, we make a bold assumption that among Asian population, rs266729 is a significant high‐risk factor for NAFLD. In different genetic models, a higher increased risk was found in homozygous model (GG vs. CC: OR = 2.13, 95% CI [1.38, 3.28]) than other four genetic models. As four studies focused on Asian population, further investigation involving diverse population should be studied in future analysis.

There were some limitations in this meta‐analysis. First, lack of detailed genotypic information and restrictions on inclusion criteria, so several studies have not been included in this analysis. Second, in some pooled analysis, there was significant heterogeneity, which may have unsatisfactorily explained using subgroup and sensitivity analyses. Third, our study only included relevant articles published in English or Chinese. In addition, gene‐environment interactions in relationship rs1501299 and rs266729 and NAFLD are worthy of consideration.

In conclusion, our meta‐analysis suggested that the rs1501299 polymorphism was not associated with NAFLD. The rs266729 polymorphism was found to be associated with a significant increase in NAFLD risk. However, more precise and larger studies will be expected to improve and perfect our findings.

## CONFLICT OF INTEREST

The authors declare no conflict of interest.
